# Evidence for Neandertal Jewelry: Modified White-Tailed Eagle Claws at Krapina

**DOI:** 10.1371/journal.pone.0119802

**Published:** 2015-03-11

**Authors:** Davorka Radovčić, Ankica Oros Sršen, Jakov Radovčić, David W. Frayer

**Affiliations:** 1 Department of Geology and Paleontology, Croatian Natural History Museum, 10000, Zagreb, Croatia; 2 Institute for Quaternary Paleontology and Geology, Croatian Academy of Science and Arts, 10000, Zagreb, Croatia; 3 Department of Anthropology, University of Kansas, Lawrence, Kansas, 66045, United States of America; University of Oxford, UNITED KINGDOM

## Abstract

We describe eight, mostly complete white-tailed eagle (*Haliaëtus* [*Haliaeetus*] *albicilla*) talons from the Krapina Neandertal site in present-day Croatia, dating to approximately 130 kyrs ago. Four talons bear multiple, edge-smoothed cut marks; eight show polishing facets and/or abrasion. Three of the largest talons have small notches at roughly the same place along the plantar surface, interrupting the proximal margin of the talon blade. These features suggest they were part of a jewelry assemblage, --- the manipulations a consequence of mounting the talons in a necklace or bracelet. An associated phalanx articulates with one of the talons and has numerous cut marks, some of which are smoothed. These white-tailed eagle bones, discovered more than 100 years ago, all derive from a single level at Krapina and represent more talons than found in the entire European Mousterian period. Presence of eight talons indicates that the Krapina Neandertals acquired and curated eagle talons for some kind of symbolic purpose. Some have argued that Neandertals lacked symbolic ability or copied this behavior from modern humans. These remains clearly show that the Krapina Neandertals made jewelry well before the appearance of modern humans in Europe, extending ornament production and symbolic activity early into the European Mousterian.

## Introduction

Ornaments are commonly associated with fossil *Homo sapiens* and are thought to represent the special cognitive abilities and symbolic capacities of modern humans [1–4]. Studies in recent years have documented Neandertals using or producing art and symbolic items evidenced by unusual lithic objects [5], feathers [6–7], modified shells [8–9], ochre [10–13], mobiliary and cave art [14–17] and very sporadically raptor talons, consisting of single elements, presumably used as pendants [18]. In the Krapina faunal collection, we have identified eight white-tailed eagle talons, suggesting that the claws from a minimum of three eagles were preserved as a group. A single phalanx was found in the same level.

Eagle talons rarely occur at European prehistoric sites and at no Neandertal site have eight been found [18]. Previously, eagle claws have been described as isolated talons at Mousterian sites stretching from about 100,000 kyr at Pech de l'Azé (FR) [6] to older than 44 kyr at Fumane cave (IT) [7]. Other examples of white-tailed eagle bones appear in European Upper Paleolithic and later sites, but they are rare and seldom involve more than one element of the foot. Presence of eight Krapina talons, four showing cut marks, suggests they were disarticulated by cutting the tendons, curated and lost as a unit, probably as a necklace or some other kind of jewelry.

The sandstone rock shelter in NW Croatia was excavated under the direction of Gorjanović-Kramberger between 1899–1905 [19–20]. Today nothing remains of the site except the eroded, sandstone cliff face. But Gorjanović-Kramberger extensively documented the site in his publications and based on the fauna estimated it to be from a warm, interglacial period, now dated to 130,000 years ago or late MIS 5e by ESR and uranium series [21]. According to Miracle [22] this age is concordant with cave bear (*Ursus spelaeus*) size and morphology. From the earliest to latest deposits the Krapina male cave bears are all small, significantly reduced in size compared to glacial sites in the region with cave bear remains. From this, Miracle suggests the strata at the site were deposited over a warm, short time period, <10,000 years [22]: “The small size of the Krapina male cave bears is best understood as an adaptation to a full interglacial climate … Krapina’s stratigraphy extended neither into later substages of the last interglacial (e.g. MIS 5a-d) nor into the last glacial MIS 3–4” (p. 85).

Only evidence of Neandertals is found at Krapina, confirmed by the appearance of Mousterian tools [23] and human remains [19–20]. Gorjanović-Kramberger and his assistant Osterman collected hundreds of Neandertal bones and teeth, more than 800 stone tools and almost 2800 animal remains [19–20, 24–25]. Everything at Krapina derived from excavations in stratified levels (S1 Fig.) and has been curated in the Croatian Natural History Museum (Zagreb) for more than a century. Animal bones as represented by minimum number of individuals (MNI), consist primarily of large mammals, especially rhinos (21.7%), bears (17.0%), and bison/bos (7.5%), while other species like pigs, deer, and small carnivores make up a lesser extent of the fauna [25]. Beavers (*Castor fiber*) are the exception, mostly coming from the fluvial base of the shelter and constitute 17.9% of the bones from site [25]. A full listing of mammalian fauna includes 25 taxa and of these 13.6% of show evidence of burning and another 4.1% preserve stone tool cut marks [25]. In addition to the mammals, Gorjanović-Kramberger recorded a turtle (*Testudo*) humerus, fresh water mussel shells of at least three taxa, several taxa of different land snail species and a few bird remains [19–20, 25]. Except for some of the bird bones, none of the non-mammalian bones or shells shows signs of human manipulation.

## Materials and Methods

Krapina specimens 385.1–385.5, 386.1–386.3, 386.18 are housed in the Department of Geology and Paleontology, Croatian Natural History Museum, Demetrova 1, 10000 Zagreb, Croatia. Surface features were first identified by eye, then further documented with a 20x microscope. Subsequent microscopic and microphotographic work was done on an Olympus DP 25 556 camera and an Olympus SZX16 microscope, using Analysis-work software. We attempted to catalog the cut marks and other surface alterations, following the criteria in Domínguez-Rodrigo et al. [26]. We found no evidence that the features we describe are the result of weathering, trampling or other natural modifications. We identify smoothing, highly polished facets and abrasion features on the talons. Smoothing occurs on the cut mark edges, so that they are not sharp as in butchering marks found experimental work [27] and on many of the other Krapina fauna [25], including the human remains [19]. We consider this smoothing the result of the talons being wrapped in some kind of fiber. Highly polished facets resemble ebrunated surfaces, where bone-on-bone contact produces a bright, shiny densely packed surface. These are small facets and must have occurred postmortem, since this region of the talon would never be in contact with another talon in life. We identify abrasion as dully-polished, surface modifications, generally on the blade of the talon. These do not reflect light like the highly polished facets described above and are presumably related to when the talons rubbed together when they were part of an assemblage. As in the polished facets, these would not have occurred in the living white-tailed eagle, since the talons are separated from each other.

## Results and Discussion

Gorjanović-Kramberger first mentioned eagle claws (“?*Aquila* Mehrere Krallen”;? golden eagle, several claws) in 1901 [28] indicating that the bones were found in the early excavations at the site. After cataloguing the material, the talons were sent to Lambrecht in Budapest, who identified them as the white-tailed eagle (*Haliaëtus albicilla)* and was the first to illustrate most of the claws [29] (S2 Fig.). In the Krapina faunal collection, there are 29 isolated bird bones (S1 Table) with eagles and owls comprising 41.4% of this sample [25]. Only the eagle bones show signs of human modification. Based on duplication of sides, we estimate they come from three-four different eagles.

Four of eight *Haliaëtus albicilla* talons and a pedal phalanx have multiple cut marks (S2 Table). One talon (385.4) bears a pencil mark, presumably made by Gorjanović-Kramberger, identifying it as coming from the uppermost level. Other talons lack pencil designations, but the bones are similar in color and preservation. The uppermost level at Krapina was called by Gorjanović-Kramberger, the “*Ursus spelaeus* zone,” due of the large number of cave bear remains found in this stratum [19–20]. There is at least one hearth and a few Mousterian tools, mainly Levallois blanks and backed knives [20, 23].

**Fig 1:**
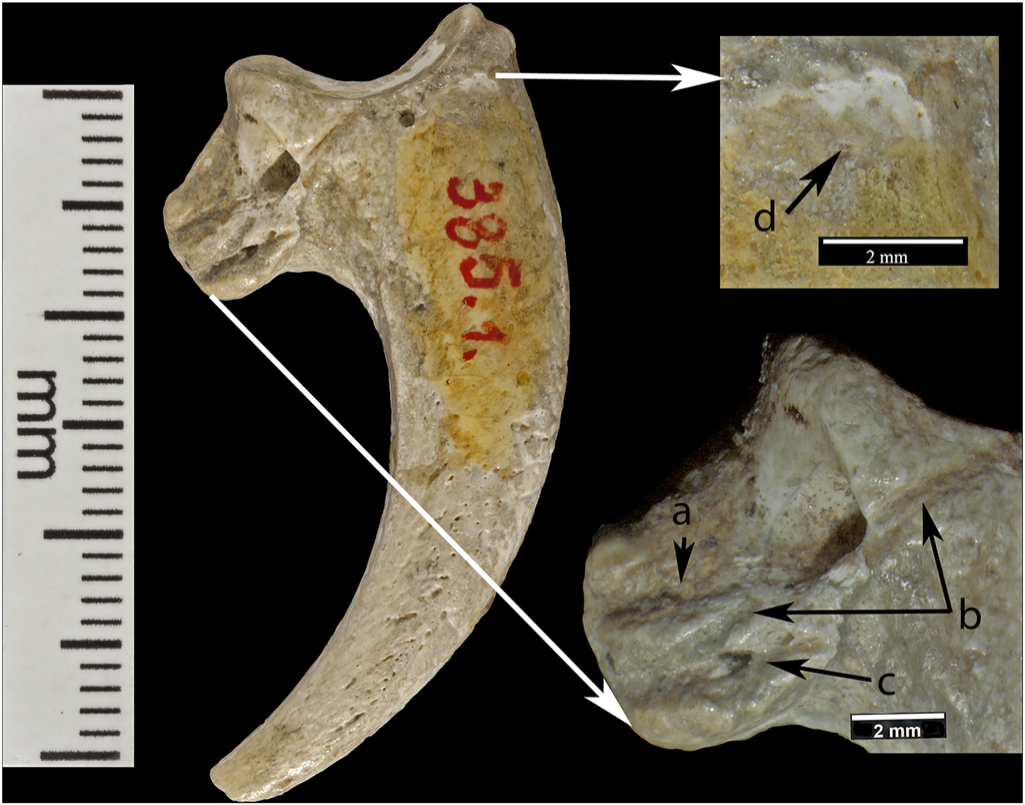
Krapina 385.1, a right talon 2. Three cut marks are preserved on the lateral surface: (a) a short superior cut mark; (b) a long cut mark interrupted by the foramen; (c) a short inferior mark. Edges of most cut marks are not sharp. An abraded area (d) occurs near the proximal edge of the joint.

The most striking *Haliaëtus albicilla* claw is Krapina 385.1, a right second talon bearing six cut marks and surface modifications. The three largest cut marks occur across the lateral side of the proximal surface, just distal to the joint with the phalanx. These marks are clearly visible in Lambrecht’s 1915 description (S2 Fig.), but were not recognized by him or Gorjanović-Kramberger even though the latter extensively documented cut marks on human and animal bones [19]. The uppermost cut mark (Fig. 1a) is the shortest (3.2mm) and is a wide, V-shaped cut. The longest cut mark begins at the joint surface where it is 3.7mm long, breaks as it extends across the foramen, then continues 4.1mm onto the projection (Fig. 1b). A third cut mark measures 5.7mm and is angled about 20° to the long cut mark (Fig. 1c). All these cut marks show edge smoothing. On the dorsal surface at the tuberculum extensorium, just perpendicular to the joint is a short (~1.0mm) cut mark. Two slightly curved, parallel cut marks (2.0mm and 1.3mm) appear about 3mm distal from the edge of the articular surface (not shown). Small areas of surface flattening and abrasion appear on the lateral, proximal margin (Fig. 1d) and more polishing occurs around the elevated surface lateral and superior to the vascular foramen.

Krapina 385.2 is a right second talon with no cut marks, but it possesses a small, abraded surface on the lateral aspect of the tuberculum flexorium major (S3 Fig.). Krapina 385.3 is a right talon 1 (or 2) with no cut marks, but has numerous areas of polish on its lateral aspect. The most impressive is a long, narrow facet beginning at the bottom third of the talon and narrows to a point 4.5mm proximal to the tip. The polished area is about 10.5mm long with a maximum width of 1.2mm at its most proximal extent (S4 Fig.).

**Fig 2:**
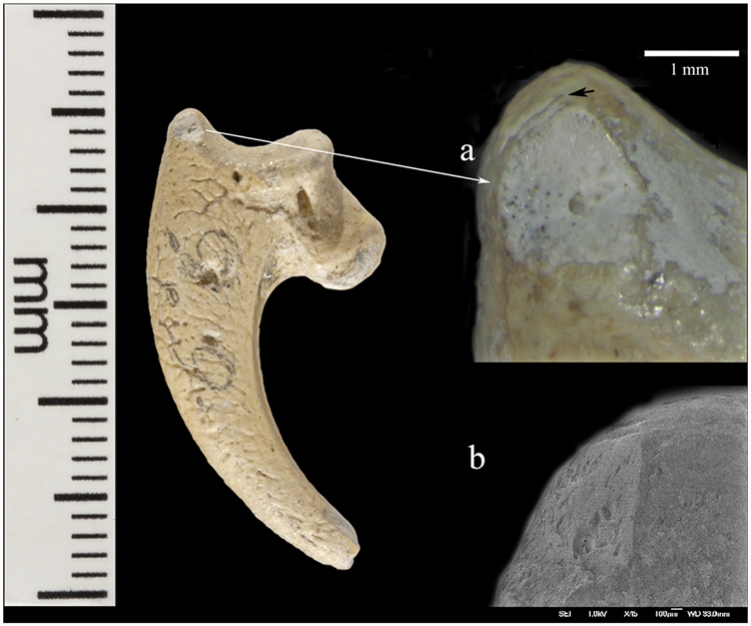
Krapina 385.4, a left talon 3. Shown are a highly polished area (a) and two cut marks (arrow) above it. A SEM (b) of the polished area is twisted ~90° from the lower magnification image showing the full face of the polished surface and the hinge fracture below it. On the talon a light pencil mark “9” is preserved at the mid-talon position.

Krapina 385.4 is a virtually intact left third talon. On the dorsal edge of the proximal joint surface are two short cut marks, paralleling each other (Fig. 2a). Lateral to these is a broad, V-shaped wedge at the border of the articular surface. The dorsal-most portion shows some polishing and compaction of this surface, which gives it an ‘ivory’ appearance (Fig. 2a-b). The inferior-most portion of this facet shows a spalled-off facet with a hinge fracture, which lacks polish. On the medial side there are two small, abraded areas, which have eroded the elevated morphology (not shown). Neither is as compact nor as shiny as the lateral facet.

**Fig 3:**
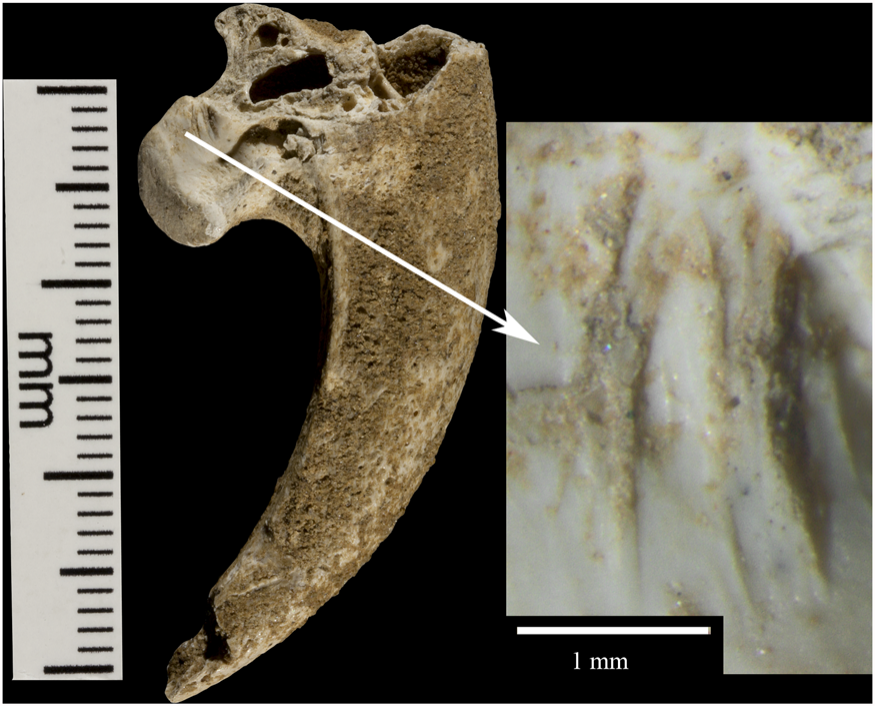
Krapina 385.5, a right talon 1. Lambrecht [29] did not include this talon (S2 Fig.), presumably because it was damaged.

Krapina 385.5 (Fig. 3) is a right talon 1, which shows a natural (excavation?) break across the base of the tuberculum flexorium major on the lateral, proximal surface. This is the only talon with large amounts of adhering cave sediment. There may be additional marks under this sediment, but well-defined cut marks occur across the ‘neck’ of the talon. Four cut marks appear on the lateral side, distal to the tuberculum flexorium. These short cut marks, off the articular surface, parallel each other and range in length from 1.3–1.6mm. The proximal is the deepest and widest compared to the others. Compared to other specimens, these cut marks show little edge smoothing.

**Fig 4:**
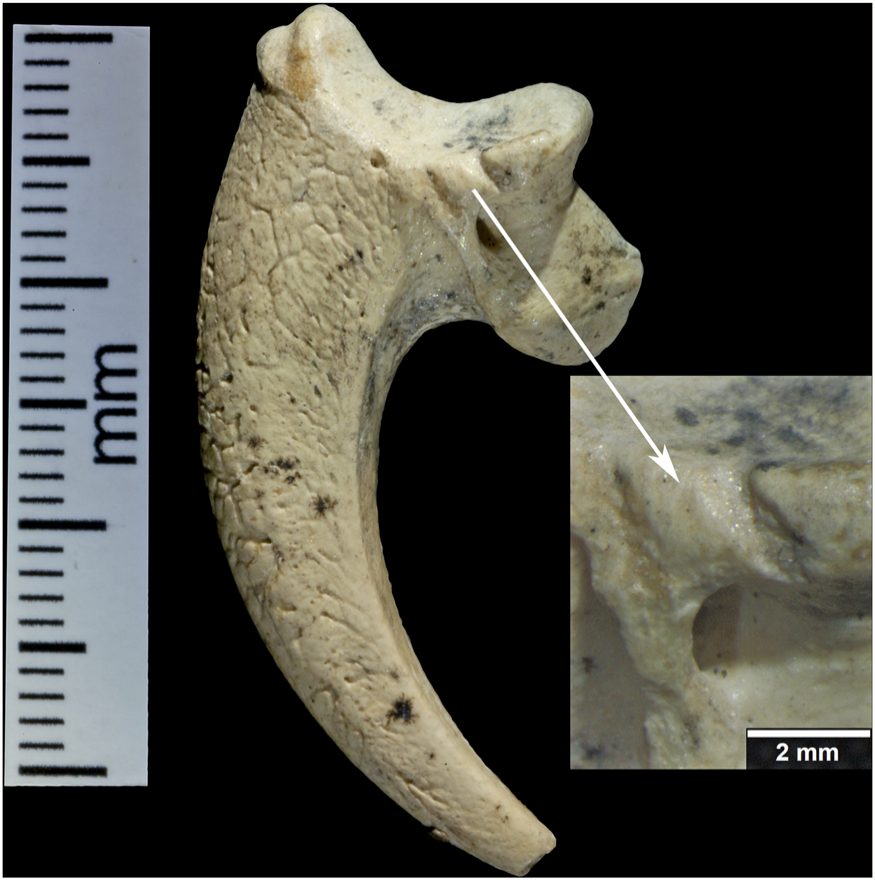
Krapina 386.1, a right talon 2. Two cut marks with smoothed edges and a magnification of the area shows that the more proximal one has the broader dimension.

A right second talon (386.1) shows cut marks along the medial articular margin (Fig. 4). Measuring 2.4mm–3.5mm, the incisions roughly parallel each other, pierce the articular surface, have some preserved cave sediment and show marginal smoothing. Like 385.3 there is a long (3.2mm) burnished facet on the lateral side, ending at the tip of the talon, which is polished (not shown). There is abrasion on the lateral aspect of the blade with a facet covered in its proximal extent by lacquer (not shown). It measures (3.4mm long with a maximum height of 1.4mm). Distal to this is a long narrow facet (3.4mm long with a maximum height of. 6mm), which narrows to the tip.

Krapina 386.2 is a left talon 1 with no cut marks, but an area of constricted polishing occurs on the medial face of the tuberculum flexorium major (S5 Fig.). Krapina 386.3 is another left third talon with a short cut mark on the superior articular wall, extending about 1.6mm into the articular facet. Just below is a small, highly polished facet on the dorsal lateral side under the lip of the articular facet and a smaller one on the ventral side (S6 Fig.). The facet is a flattened surface, similar to the one found on 385.4. Diagonally, on the medial side there is a small crescent facet positioned at the medial ventral portion of the articular lip.

**Fig 5:**
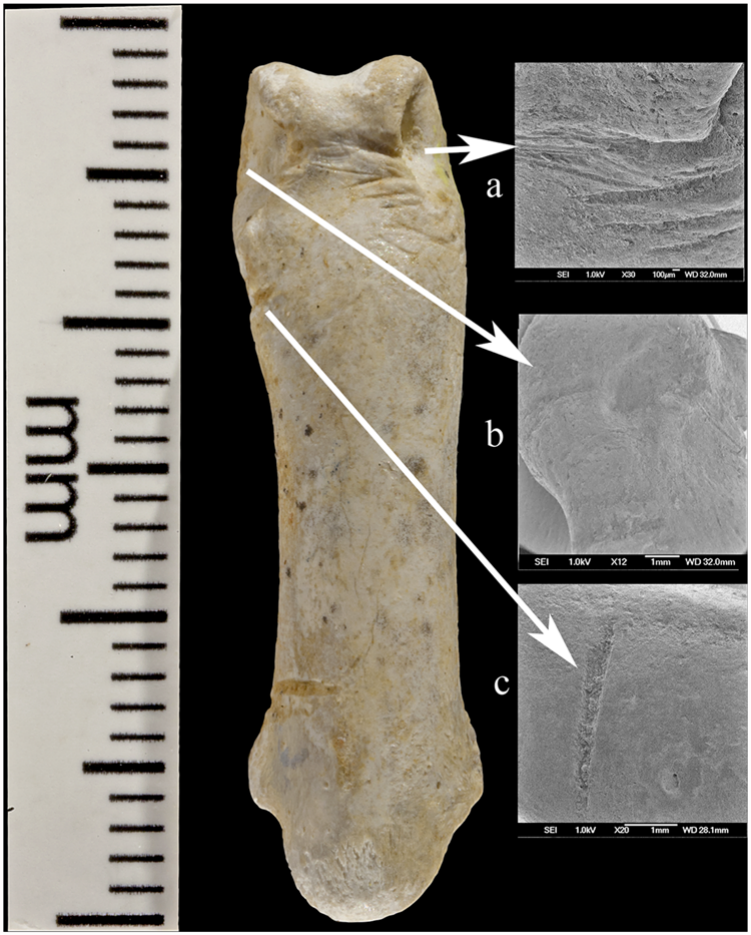
Krapina 385.18, a left digit 3 phalanx with multiple cut marks. SEMs include (a) distal cut marks on the dorsal, (b) cut marks on the diaphysis at the distal, lateral edge and (c) a distal, lateral cut mark on the diaphysis.

**Fig 6:**
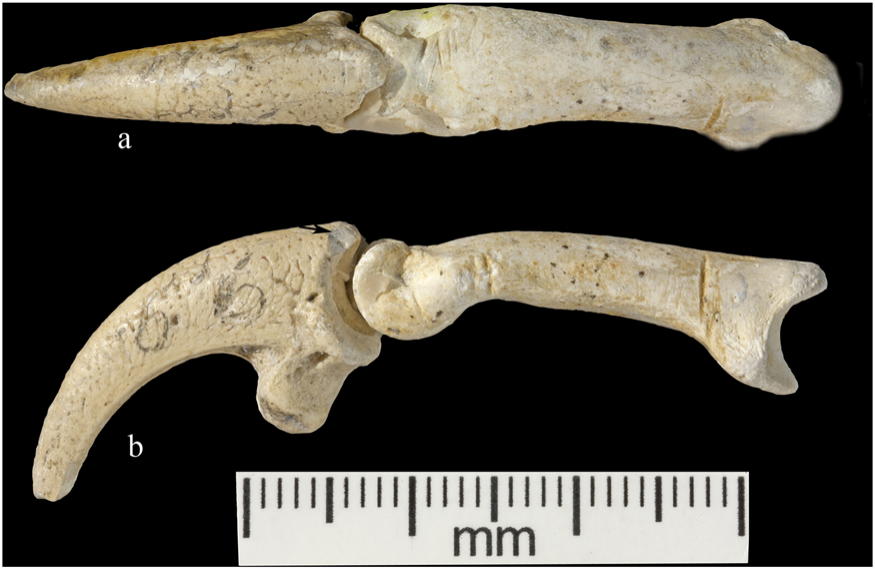
Articulation of Krapina 385.4 and 386.18. Dorsal (a) and lateral (b) view; arrow in (b) points to highly polished area on 385.4.

Krapina 386.18 is an intact third phalanx of digit 3. It was originally assigned to *Aquila* [30], then to *Bubo bubo* [31]. Our comparisons with equivalent phalanges of modern *Bubo bubo* and *Haliaëtus albicilla* indicate it is a white-tailed eagle. The phalanx is light in color and similar in preservation to the other Krapina eagle talons. At least 21 cut marks appear on the lateral and dorsal surfaces (Fig. 5a-c). The most prominent wraps from the lateral to dorsal face at the proximal end. About 5.0mm long, the incision deeply penetrates the cortical bone and the interior of the mark is stained with cave sediment. It is flanked proximally by another distinct mark 4.0mm long, which curves from the plantar to the dorsal edge. Distal to this cut mark are a series of very shallow, faint incisions on the lateral edge. These range in length from 1.6–2.1mm. Continuing more distal, shorter and deeper cut marks are found proximal to the condyle and another two appear on the lateral wall of the condyle. All measure about 2.5mm. Dorsally, cut marks are only found on the distal region across the bone separating the two dorsal fossae. In this location at least six marks pierce the bone, varying in length, width and depth of penetration into the bone. The longest (2.1mm) and widest (.3mm) is at the medial end and is flanked by a distinctive, more proximal cut mark. In these, there is some smoothing of the cut mark edges. The remaining marks are fainter, but have sharper edges. There are no cut marks on the plantar or medial surfaces (S7 Fig.). This terminal phalanx articulates with the left talon 3 (385.4), so the two bones are likely from the same individual (Fig. 6).

Miracle [25], describing the animal remains from Krapina, recorded a high percentage of bones and teeth deriving from large mammals. By MNI birds make up less than 4% of the assemblage and in most cases are represented by isolated specimens (S1 Table). This may relate to the size and fragility of the bones or to collector bias, but in the existing avifauna from Krapina there is no evidence that birds were being consumed as in other Middle Paleolithic sites [32–33]. For the limited bird remains, most of the non-raptor species are isolated bones and could have entered the sediments without human involvement. This is very unlikely for the white-tailed eagle talons. Modern ornithologists report in central and eastern Croatia that white-tailed eagles build large nests in trees, on cliffs or occasionally on the ground [34], so it is likely the birds were acquired elsewhere and brought to the site.

White-tailed eagles are impressive birds with aggressive personalities and are not easy to catch or trap, today or in the past [35–36]. Modern ones have a two-meter wingspan, a body weight from 3.0–6.5 kgs [37] and are the top diurnal, avian predators in Europe today. Based on talon size, white-tailed eagles at Krapina were similar in body size. Compared to other avian species, they are relatively rare in the environment, yet their bones represent the majority of the avifauna sample at Krapina.

Cut marks on these eagle bones fit the pattern of stone tool striations as defined by Domínguez-Rodrigo et al. [26]. The deposits at Krapina were sandy and in some cases the bones were soft, making trampling or other post-depositional factors a possible explanation for the talon modifications. As shown in the above figures, most cut marks on the Krapina eagle bones are straight, deep V-shaped grooves located in specific areas in the proximal aspects of the talon. We could not evaluate all the categories compiled by Domínguez-Rodrigo et al. [26], but in addition to those above, we note that the marks on the talons are localized, never occurring across the blade of the talon or on the plantar surface. On the single phalanx, there are many cut marks, which are more characteristic of human-made cut marks than trampling. These cut marks are straight-walled, V-shaped, and often parallel each other. Some of the cut marks preserve sandy cave sediment (Fig. 5c) and, like the talons, many of the cut mark edges show smoothing. For the phalanx the cut marks are widespread, but are never on the plantar surface or medial wall and are more common in the proximal and distal ends than the midsection of the bone. Overall, the bone surfaces lack fine striations, typical of trampling. For the talons and the phalanx, we think there is no doubt that they were produced by Neandertal manipulation.

The Krapina human remains found lower in the sequence sometimes have cut marks, but these mainly are sharp-edged and seldom show signs of edge smoothing [19]. The hominid collection is extensively fragmented [24], unlike the eagle talons, which are essentially intact. None of the human remains have densely polished facets or abraded areas like on the talons, but a few the patellae show arthritic, ebrunated surfaces, the result of cartilage breakdown and arthritis. This would not be a likely cause of the polished facets on the talons, but suggests some kind of prolonged contact with another hard surface.

Miracle has extensively documented taphonomic features of the vertebrate fauna and notes that “modification of bone surfaces is extremely rare … [with] only 6.6% of the vertebrate fauna showing evidence of weathering,” p. 236 [25]. Based on his observations and our descriptions of the cut marks and other damages to the talons, there is no evidence that diagenetic factors could have produced the modifications on the eagle talons. We have also surveyed the nonhuman vertebrate faunal remains from levels 8–9 and found no evidence for similar bone modifications, other than the occurrence of cut marks. Review of faunal elements from lower levels at Krapina show no surface modifications like found on the eagle talons. Finally, there are a few other bird remains from Krapina (S1 Table) and, while broken, none show cut marks or other indications of human manipulation.

**Fig 7:**
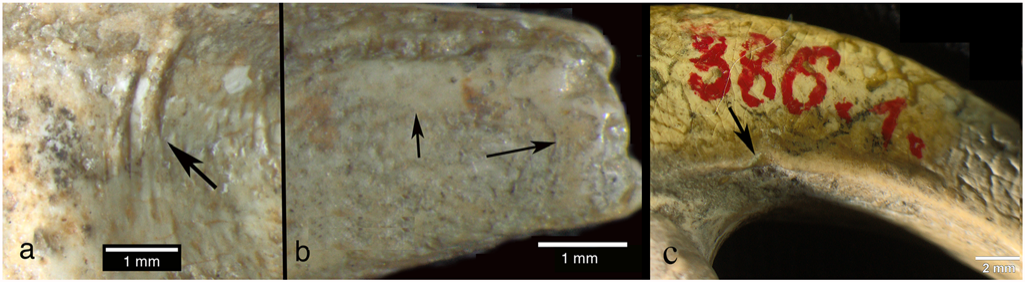
Three examples of human manipulation. (a) Smoothed cut marks on the articular facet of 385.1; (b) burnished area near the tip of 386.1; (c) nick on the otherwise sharp plantar margin of 386.1. The nick is partly filled with varnish, applied when the specimen was inventoried in the early 1900s.

Virtually all of the talons show some evidence of cut mark edge smoothing and surface abrasion at various places on the talon and three have nicks in the medial and/or lateral blade margin. (1) Cut mark smoothing. Compared to other eagle talons documented from Mousterian sites, like Mandrin cave where the cut marks have sharp edges [27], most cut mark edges of the Krapina talons are smoothed (Fig. 7a). Many of the marks are located in positions, which have been reproduced in experimental butchering [27], but subsequent, localized edge alterations appear in the Krapina specimens. Other parts of the same talon show no surface modifications, indicating that the edge wear was focused in its occurrence. (2) Abrasion. All eight talons exhibit shiny surfaces, ranging from small areas of dense, highly polished areas on the proximal ends to longer, shiny areas on the distal talon blades (Fig. 7b). They are always localized, never appear on the plantar aspects of the talons and seem to be the result of repetitive contact with another object. They could not have been produced in the living white-tailed eagle. (3) Nicks. In three of the largest talons (385.1, 385.3, 386.1) small nicks interrupt the sharp edge of the blade’s medial and/or lateral margins. Sometimes these appear on both margins (385.3 and 386.1) or on only the medial margin (385.1) (Fig. 7c). They are located about 10mm distal to the articular margin. We cannot rule out taphonomic causes for these indentations into the sharp medial and lateral talon edges, but they are limited in occurrence and are never found elsewhere on the talons or on any of the smaller talons. In contrast the other five talons clearly show sharp, uninterrupted edges all along the blade margins.

For the single phalanx there are only cut marks over the dorsal and lateral surfaces. These appear mainly on the proximal and distal ends, while most of the diaphysis, the medial and the plantar surfaces are free of any manipulation evidence. Like in the talons, many of the cut mark edges tend to be smoothed, but a few show sharp, unmodified margins (see Fig. 5).

Some of these features have not been documented in single talons found at other Mousterian sites, possibly because they were suspended as pendants with no neighboring abrasive surfaces. While on a different medium, in some respects, the abrasion on the talons resembles what has been described as “use wear” surfaces in the shells from Blombos in S. Africa. In *Nassarius kraussianus* specimens polished facets occur on the margins of holes on the shells. Henshilwood et al. [38] interpreted these as facets produced when the shells rubbed against each other from being mounted on a string. Vanhaeren et al. [39] reproduced edge wear facets by stringing shells together, shaking them and found that edge wear only occurred when “the string was soaked in a solution of diluted vinegar mixed with ochre powder … and the shells were in direct contact with each other.” From these experiments, they contended that an acidic environment, coming from sweat or urine, was essential for producing the polishing. We have not done experimental archaeology or chemical analyses on these or other eagle talons and there are no visible traces of ochre on the talons. While on shell, not talons, following the Blombos bead work [39] we propose the densely polished and burnished areas are the result of the talons being strung. Then they rubbed against each other to produce the abrasions. We assume that smoothing of cut mark edges is related to them being tied with a string or sinew. We know Neandertals made twisted fibers at one site [40] and there is no reason to suspect the Krapina Neandertals were incapable of modifying fibers to produce a twisted fiber or a sinew filament for stringing together eagle talons.

## Conclusions

Eagle talons are rare at other Neandertal localities and no sites have yielded eight talons from white-tailed eagles or any other raptor. Since three-four different eagles are represented, they must have been acquired in separate events and were preserved as a unit before they were lost in the sediments. Others have noted [6–7, 27, 41–43] that raptor bones found in late Pleistocene sites signal some kind of symbolic activity. At Krapina, cut marks on the pedal phalanx and talons are not related to feather removal or subsistence, so these must be the result of severing tendons for talon acquisition. Further evidence for combining these in jewelry is edge smoothing of the cut marks, the small polished facets, medial/lateral sheen and nicks on some specimens. All are a likely manifestation of the separating the bones from the foot and the attachment of the talons to a string or sinew. Cut marks on many aspects, but not the plantar surfaces, illustrate the numerous approaches the Neandertals had for severing the bones and mounting them into a piece of jewelry.

As in ethnohistoric-present societies, the Neandertals’ practice of catching eagles very likely involved planning and ceremony [35]. We cannot know the way they were captured, but if collected from carcasses it must have taken keen eyes to locate the dead birds as rare as they were in the prehistoric avifauna. We suspect that the collection of talons from at least three different white-tailed eagles mitigates against recovering carcasses in the field, but more likely represents evidence for live capture. In any case, these talons provide multiple new lines of evidence for Neandertals’ abilities and cultural sophistication. They are the earliest evidence for jewelry in the European fossil record and demonstrate that Neandertals possessed a symbolic culture long before more modern human forms arrived in Europe.

## Supporting Information

S1 Fig Stratigraphic column at Krapina. Gorjanović-Kramberger and his assistant Osterman excavated Krapina in levels. Shortly after 1905, the site was completely emptied of all sediments, so only the eroded back of the site exists today. Gorjanović-Kramberger commissioned an “engineer Kos” to draw the stratigraphy shown here, which other than some sketches in Gorjanović-Kramberger’s field notes is the only existing drawing of the site [20]. The uppermost part of the sequence (layers 8/9) was identified as the *Ursus spelaeus* zone. From his description, we know the chronological position of all the white-tailed eagle fauna is from the top of the Krapina geological sequence [20], designated by the arrows. Most human remains come from level 4, the so-called *Homo sapiens* zone, but a few were discovered in the upper part of the Krapina sequence [19]. Based on sedimentation rates and the spread of absolute dates, the Krapina chronological time span is short, less than 10,000 years. In the drawing, “a” represents the Miocene sandstone forming the shelter’s walls and ceiling and “b” the fill.
(TIF)

S2 Fig Lambrecht’s illustration of the talons. The first image of seven of the eight Krapina talons was published by Lambrecht, who correctly identified the talons as coming from *Haliaëtus albicilla*. Krapina 385.1 is on the top row left and the three cut marks running across the tuberculum flexorium major are clearly visible (arrow). On the lower row, second from right, is Krapina 385.4 and its polished facet is apparent (arrow). The image is from Lambrecht [44], but identical to his 1915 article [29]. We added the arrows.
(TIF)

S3 Fig Medial and lateral views of Krapina 385.2, a right talon 1. (TIF)

S4 Fig Medial and lateral views of Krapina 385.3, a right talon 2. The arrows indicate a long burnished area on the distal lateral border.
(TIF)

S5 Fig Medial and lateral views of 386.2, a left talon 1. (TIF)

S6 Fig Medial and lateral views of Krapina 386.3, a left talon 3. Arrows (a-b) point to two small highly polished areas.
(TIF)

S7 Fig Medial (a) and plantar (b) views of 386.18. Note the absence of cut marks, compared to the highly modified dorsal and lateral surfaces (e.g., Figs. 5–6 in text)
(TIF)

S1 Table Other bird remains at Krapina. Their stratigraphic association is unclear and bird remains are very limited in the faunal collection, consisting of single bones. All are fragmentary and none show signs of human manipulation. Most of these were identified by Malez and Malez [30–31].
(DOC)

S2 Table Inventory of eagle talons and phalanx with the type of human manipulation. (DOC)
