# Supplementary figures and images for: Evidence for Neandertal Jewelry: Modified White-Tailed Eagle Claws at Krapina
Source: PLoS One. 2015 Mar 11;10(3):e0119802. doi: 10.1371/journal.pone.0119802 (PMC4356571; doi:10.1371/journal.pone.0119802)

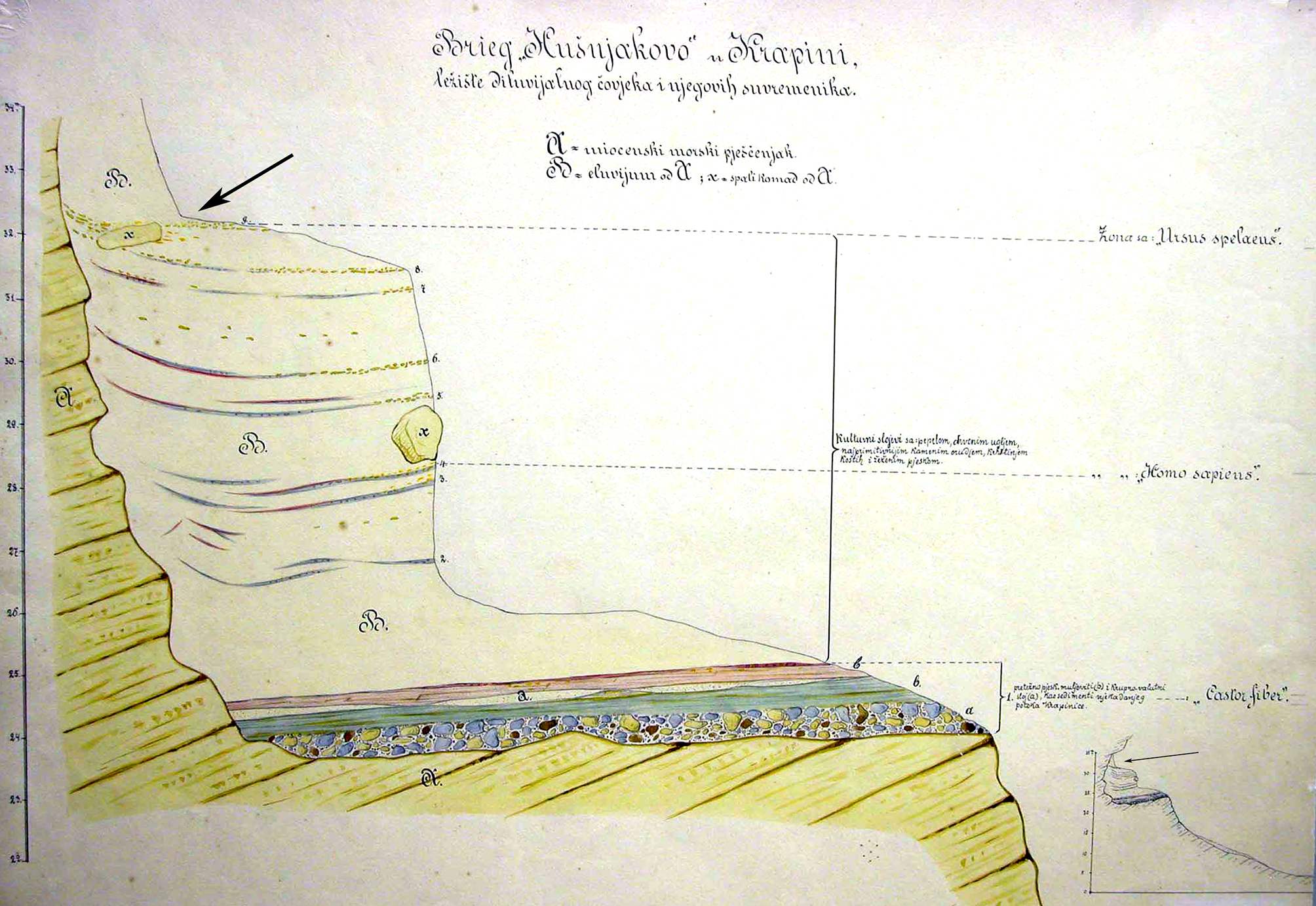

Supplement: S1 Fig — Gorjanović-Kramberger and his assistant Osterman excavated Krapina in levels. Shortly after 1905, the site was completely emptied of all sediments, so only the eroded back of the site exists today. Gorjanović-Kramberger commissioned an “engineer Kos” to draw the stratigraphy shown here, which other than some sketches in Gorjanović-Kramberger’s field notes is the only existing drawing of the site [20]. The uppermost part of the sequence (layers 8/9) was identified as the Ursus spelaeus zone. From his description, we know the chronological position of all the white-tailed eagle fauna is from the top of the Krapina geological sequence [20], designated by the arrows. Most human remains come from level 4, the so-called Homo sapiens zone, but a few were discovered in the upper part of the Krapina sequence [19]. Based on sedimentation rates and the spread of absolute dates, the Krapina chronological time span is short, less than 10,000 years. In the drawing, “a” represents the Miocene sandstone forming the shelter’s walls and ceiling and “b” the fill. (TIF) [file pone.0119802.s001.tif]

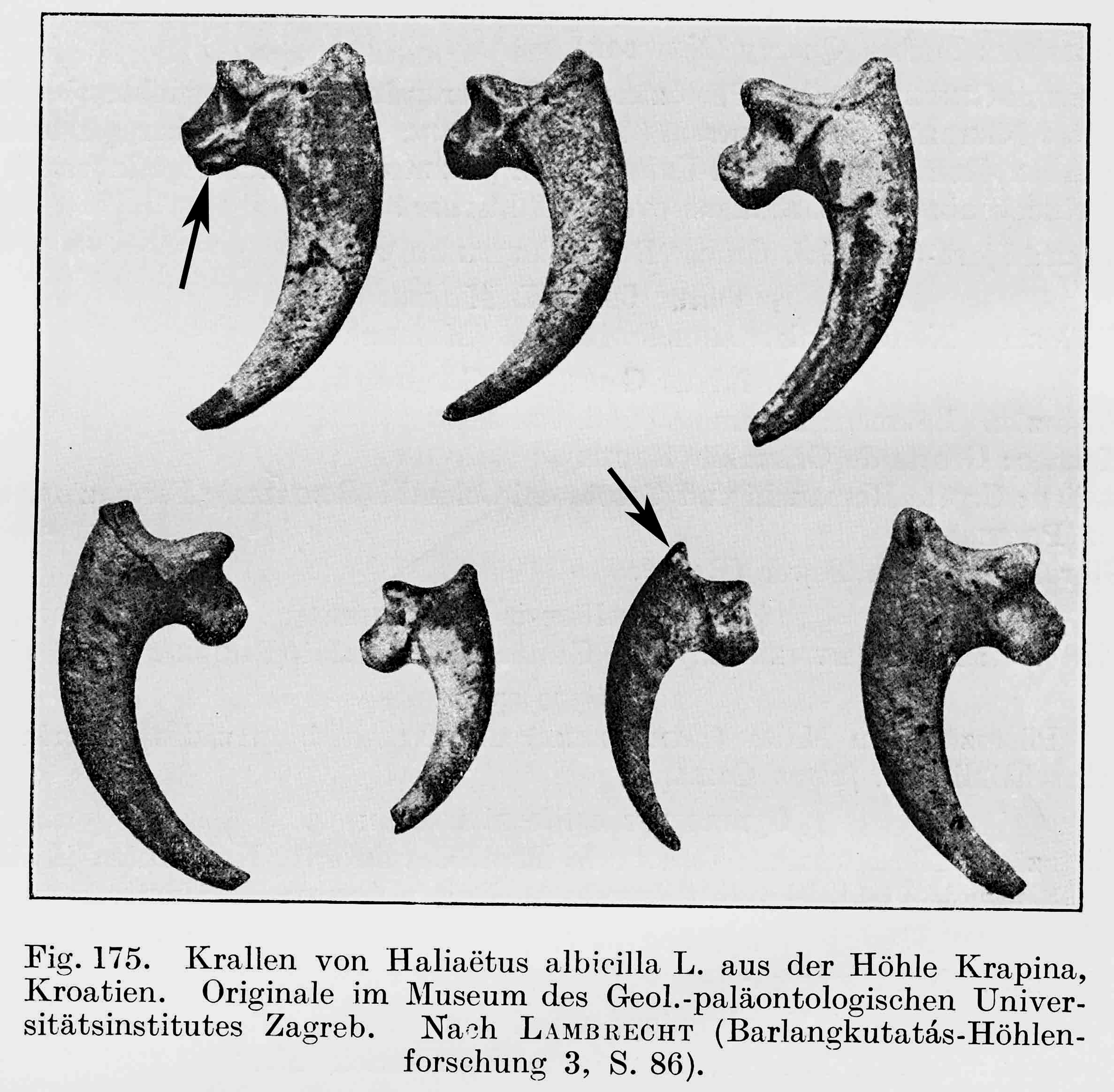

Supplement: S2 Fig — The first image of seven of the eight Krapina talons was published by Lambrecht, who correctly identified the talons as coming from Haliaëtus albicilla. Krapina 385.1 is on the top row left and the three cut marks running across the tuberculum flexorium major are clearly visible (arrow). On the lower row, second from right, is Krapina 385.4 and its polished facet is apparent (arrow). The image is from Lambrecht [44], but identical to his 1915 article [29]. We added the arrows. (TIF) [file pone.0119802.s002.tif]

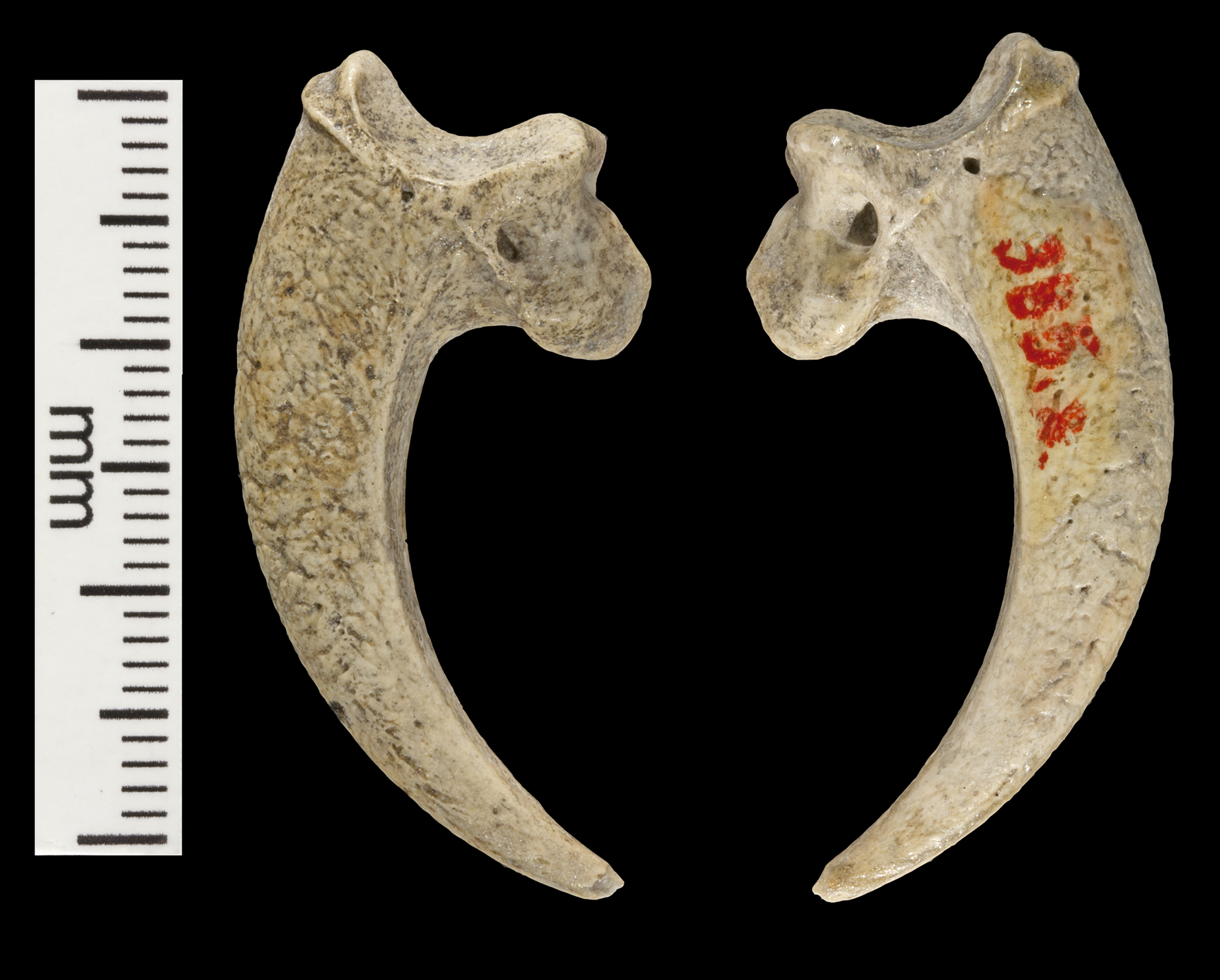

Supplement: S3 Fig — (TIF) [file pone.0119802.s003.tif]

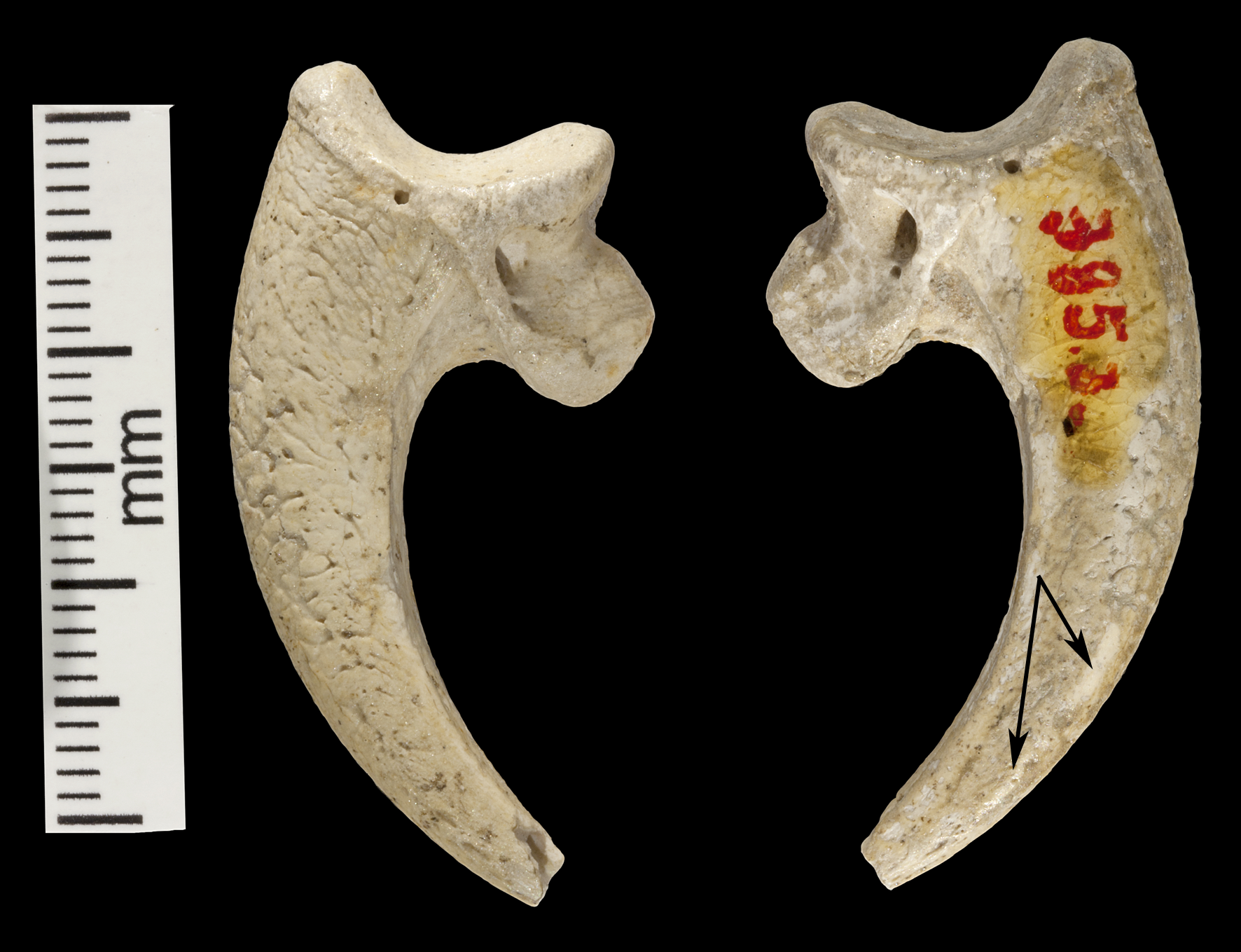

Supplement: S4 Fig — The arrows indicate a long burnished area on the distal lateral border. (TIF) [file pone.0119802.s004.tif]

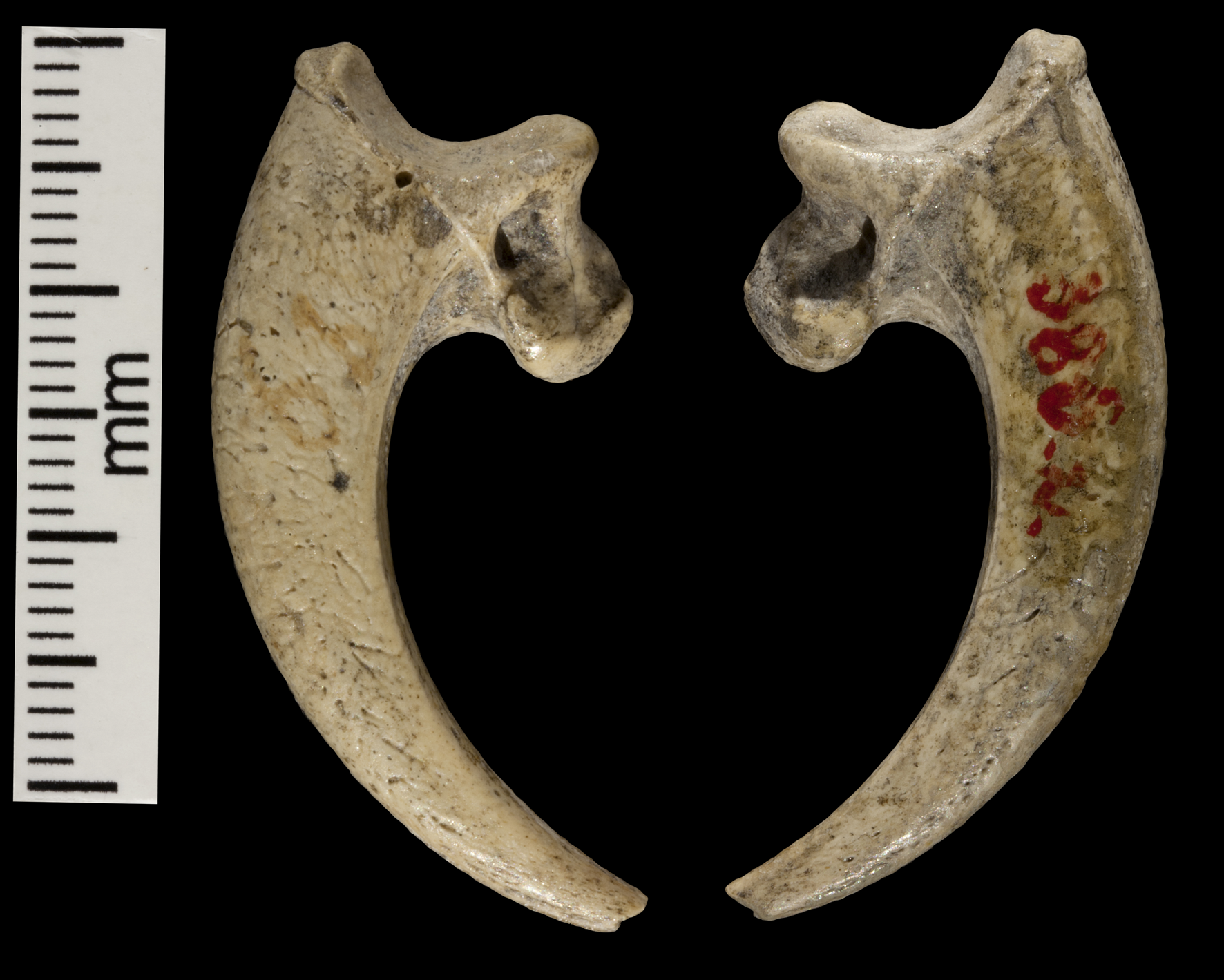

Supplement: S5 Fig — (TIF) [file pone.0119802.s005.tif]

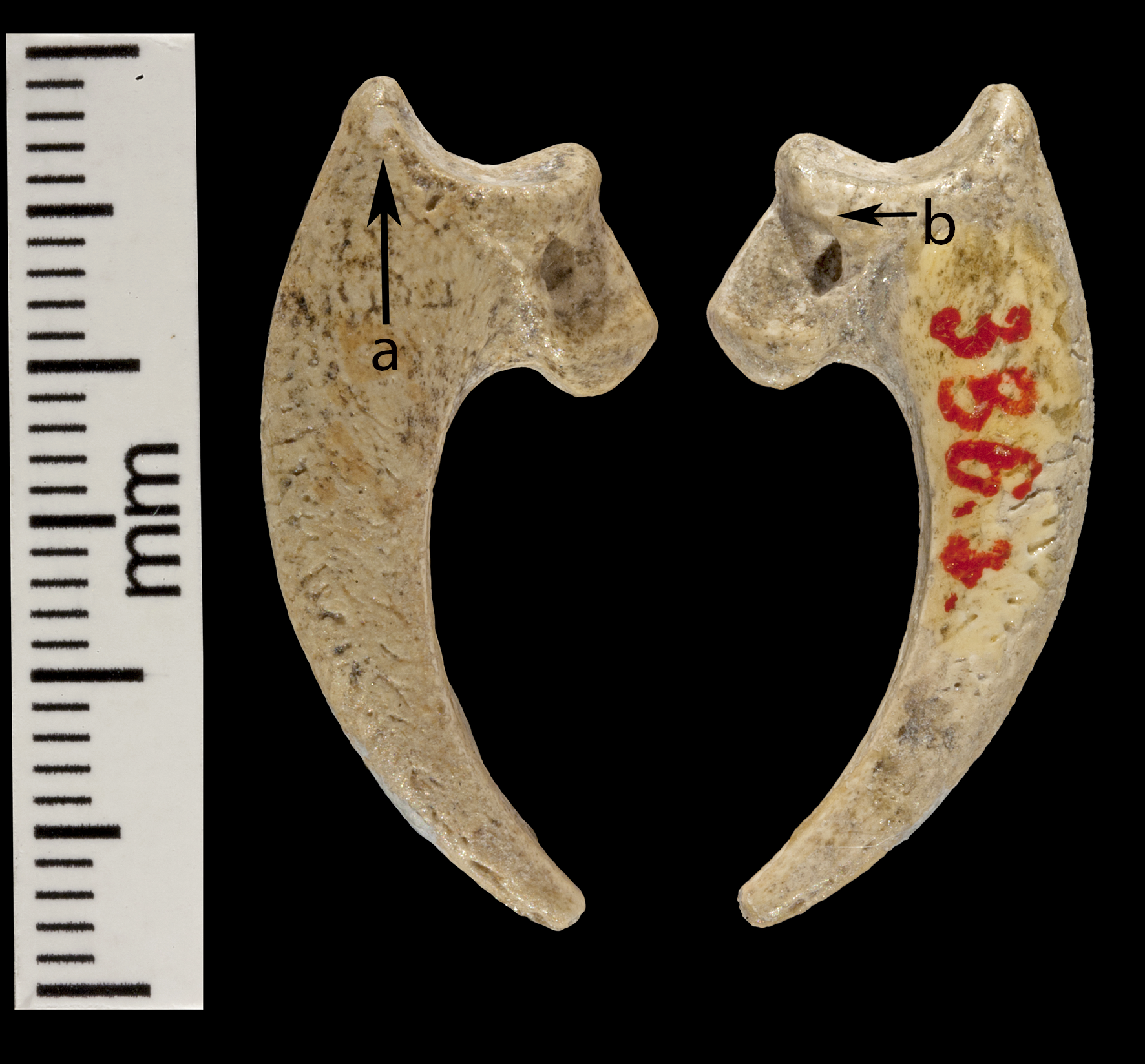

Supplement: S6 Fig — Arrows (a-b) point to two small highly polished areas. (TIF) [file pone.0119802.s006.tif]

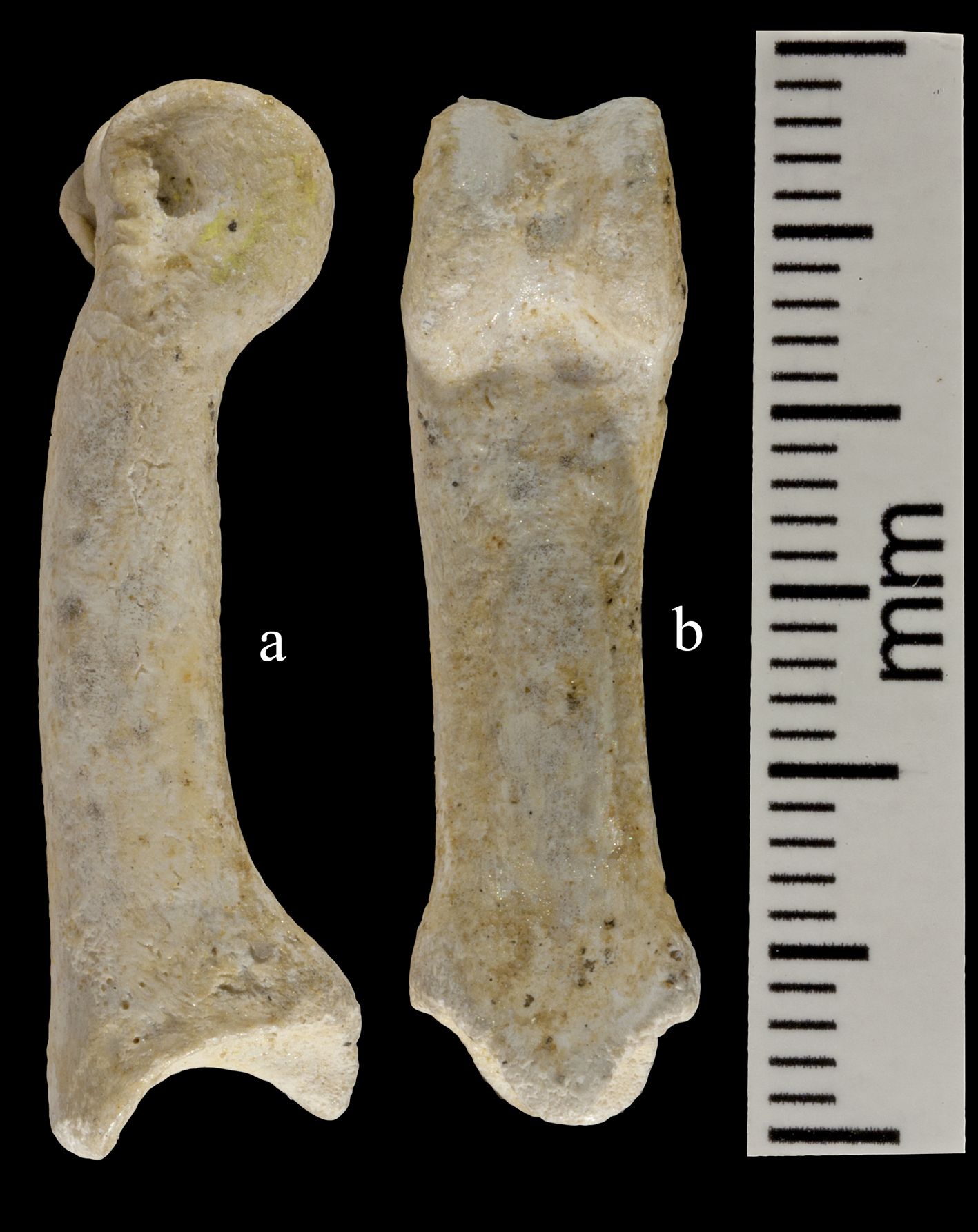

Supplement: S7 Fig — Note the absence of cut marks, compared to the highly modified dorsal and lateral surfaces (e.g., Figs. 5–6 in text) (TIF) [file pone.0119802.s007.tif]
