# Supplementary material for: Evidence for Neandertal Jewelry: Modified White-Tailed Eagle Claws at Krapina
Source: PLoS One. 2015 Mar 11;10(3):e0119802. doi: 10.1371/journal.pone.0119802 (PMC4356571; doi:10.1371/journal.pone.0119802)
Supplement: S1 Table — Their stratigraphic association is unclear and bird remains are very limited in the faunal collection, consisting of single bones. All are fragmentary and none show signs of human manipulation. Most of these were identified by Malez and Malez [30–31]. (DOC) [file pone.0119802.s008.doc]

**S1 Table. Other bird remains at Krapina^a^**

| Family (Order) | Species | Common name | Element |
| --- | --- | --- | --- |
| Anatidae | *Aythya* cf*. fuligula* | Tufted Duck | tarsometatarsus |
| Accipitridae | *Gyps fulvus* | Eurasian Griffon | sternum |
| Tetraonidae | *Bonasa bonasia* | Hazel Grouse | left coracoid, right coracoid, right coracoid, left femur, right humerus |
| Phasianidae | *Perdix perdix* | Grey Partridge | left carpometacarpus |
| Strigidae | *Strix aluco* | Tawny Owl | right carpometacarpus |
| Turdidae | *Turdus viscivorus* | Mistle Thrush | right humerus, right femur, right femur, left tibiotarsus |
| Turdidae | *Turdus merula* | Common Blackbird | left and right humerus |
| Corvidae | *Garrulus glandarius* | Acorn Jay | left coracoid |
| (Passeriformes) | Unknown | - | humerus  tibiotarsus |

^a^Together with 11 *Haliaëtus albicilla* bones (tibia, fibula, 8 talons and a phalanx) - a total of 29 Krapina bird remains.
