# Supplementary material for: Evidence for Neandertal Jewelry: Modified White-Tailed Eagle Claws at Krapina
Source: PLoS One. 2015 Mar 11;10(3):e0119802. doi: 10.1371/journal.pone.0119802 (PMC4356571; doi:10.1371/journal.pone.0119802)
Supplement: S2 Table — (DOC) [file pone.0119802.s009.doc]

| Krapina specimen number | talon | Modification type | | |
| --- | --- | --- | --- | --- |
|  |  | cut marks | abrasions | nicks^a^ on  talon edge |
| 385.1 | right 2 | 🗸 | 🗸 | 🗸 |
| 385.2 | right 2 |  | 🗸 |  |
| 385.3 | right 1 (or 2) |  | 🗸 | 🗸 |
| 385.4 | left 3 | 🗸 | 🗸 |  |
| 385.5 | right 1 | 🗸 | 🗸 |  |
| 386.1 | right 2 | 🗸 | 🗸 | 🗸 |
| 386.2 | left 1 |  | 🗸 |  |
| 386.3 | left 3 |  | 🗸 |  |
| phalanx | |  | | |
| 386.18 | left digit 3 | 🗸 | 🗸 |  |

**S2 Table Inventory of eagle talon and phalanx with type of human manipulation**

^a^Note on the nicks. Nicks are present only on the largest talons at approximately the same distance from the articular surface, always cutting the sharp plantar margin of the body. Talons from living eagles have distinctly sharp plantar margins and these areas in live animals are protected with thick keratinous layer.
